# Are Pathogenic *Leptospira* Species Ubiquitous in Urban Recreational Parks in Sydney, Australia?

**DOI:** 10.3390/tropicalmed9060128

**Published:** 2024-06-06

**Authors:** Xiao Lu, Mark E. Westman, Rachel Mizzi, Christine Griebsch, Jacqueline M. Norris, Cheryl Jenkins, Michael P. Ward

**Affiliations:** 1 Sydney School of Veterinary Science, The University of Sydney, Sydney, NSW 2006, Australia; xiao.lu@sydney.edu.au (X.L.); mark.westman@dpi.nsw.gov.au (M.E.W.); christine.griebsch@sydney.edu.au (C.G.); jacqui.norris@sydney.edu.au (J.M.N.); 2 Elizabeth Macarthur Agricultural Institute (EMAI), Woodbridge Road, Menangle, NSW 2568, Australia; rachel.mizzi@dpi.nsw.gov.au (R.M.); cheryl.jenkins@dpi.nsw.gov.au (C.J.)

**Keywords:** leptospirosis, environmental, epidemiology, canine, public health, one health, soil, water, veterinary science

## Abstract

Leptospirosis is a zoonotic disease caused by the spirochete bacteria *Leptospira* spp. From December 2017 to December 2023, a total of 34 canine leptospirosis cases were reported in urban Sydney, Australia. During the same spatio-temporal frame, one locally acquired human case was also reported. As it was hypothesised that human residents and companion dogs might both be exposed to pathogenic *Leptospira* in community green spaces in Sydney, an environmental survey was conducted from December 2023 to January 2024 to detect the presence of pathogenic *Leptospira* DNA in multipurpose, recreational public parks in the council areas of the Inner West and City of Sydney, Australia. A total of 75 environmental samples were collected from 20 public parks that were easily accessible by human and canine visitors. Quantitative PCR (qPCR) testing targeting pathogenic and intermediate *Leptospira* spp. was performed, and differences in detection of *Leptospira* spp. between dog-allowed and dog-prohibited areas were statistically examined. The global Moran’s Index was calculated to identify any spatial autocorrelation in the qPCR results. Pathogenic leptospires were detected in all 20 parks, either in water or soil samples (35/75 samples). Cycle threshold (Ct) values were slightly lower for water samples (Ct 28.52–39.10) compared to soil samples (Ct 33.78–39.77). The chi-squared test and Fisher’s exact test results were statistically non-significant (*p* > 0.05 for both water and soil samples), and there was no spatial autocorrelation detected in the qPCR results (*p* > 0.05 for both sample types). Although further research is now required, our preliminary results indicate the presence of pathogenic *Leptospira* DNA and its potential ubiquity in recreational parks in Sydney.

## 1. Introduction

Leptospirosis is a zoonotic disease caused by spirochete bacteria of the genus *Leptospira* [1]. The genus consists of two clades and four subclades, with at least 64 genomically distinct species [2]. The pathogenic *Leptospira* organisms (clade P, including two subclades of pathogenic and intermediate pathogenic *Leptospira*) could potentially infect many mammalian species, including dogs and humans. In reservoir hosts, *Leptospira* causes asymptomatic, chronic infection, replicates in the host’s proximal renal tubules, and is shed in the urine [3]. Theoretically, all mammalian species can act as reservoirs for *Leptospira*, but the most important reservoir species in human environments are rodents [1]. While chronic infection and shedding occur in humans and dogs [4,5], leptospirosis in these two species is more likely to be acute and life-threatening (incidental hosts) [1,3,6]. Infection occurs through direct contact with infected kidney tissues or urine or indirectly from contaminated environmental sources (most commonly water and soil) [7]. *Leptospira* spp. are known for their resilience in the environment post-excretion, with reported survival times in open natural and sterile experimental environments of several months [8,9]. The multiplication of *Leptospira* spp. in poorly drained, moist soil has been observed [10].

In humans, leptospirosis is a disease highly associated with extreme meteorological events and occupational exposure. In both developing and developed countries, outbreaks of human leptospirosis are frequently observed following heavy rainfall and flooding [11,12,13,14,15,16], with the hypothesis that pathogenic *Leptospira* organisms inhabit soil and water sediments at various depths prior to being released by excessive erosion [17]. Occupations that are directly exposed to mammalian reservoirs (e.g., rodents and cattle) or indirectly exposed to contaminated water and soil are at a higher risk of leptospirosis (e.g., farmers, abattoir workers, construction workers, and veterinarians) [18,19,20]. Some outdoor recreational activities (e.g., camping, whitewater rafting, swimming, and caving) have also been identified as risk factors for leptospirosis [21,22,23,24]. Leptospirosis in dogs involves the same direct and indirect transmission pathways, and thus infection is also associated with flooding events [25], reservoir hosts, and environmental exposure related to the purpose of the animal (e.g., hunting or herding dogs in rural areas) [26,27]. Although leptospirosis was traditionally considered a rural disease, recent studies have described a shift in both human and canine patient demographics to “less-exposed” groups living in urban areas [23]. However, little research has been performed into the epidemiology of *Leptospira* in highly human-modified, metropolitan areas with high income and population density.

In Sydney, New South Wales (NSW), Australia, both locally acquired canine and human leptospirosis cases were not reported for decades [28,29,30]. Unlike human leptospirosis, canine leptospirosis is not a notifiable disease in the state of NSW [31]. Since 2017, canine leptospirosis has apparently re-emerged with veterinarians voluntarily sharing information about confirmed canine leptospirosis cases in urban Sydney via personal communication [28]. As of January 2024, the number of veterinarian-reported canine leptospirosis cases have risen to 34. These cases have not shown significant seasonality, and no association with local climate (temperature and rainfall) has been found [32]. The clinical cases were correlated with canopy coverage in the neighbourhood, but not with the presence of recreational areas in the vicinity [32]. In 2021, a locally acquired case of human leptospirosis was reported. This patient had a history of working at a recreational golf course in eastern urban Sydney [30]. This human case, together with a previously identified correlation between landscape factors and cases of canine leptospirosis, indicates possible environmental exposure of humans and dogs to pathogenic *Leptospira* in urban green spaces in Sydney and the potential ubiquity of the pathogen across the city. To test these two hypotheses, a focused environmental survey of pathogenic *Leptospira* in urban green spaces (e.g., recreational parks) is required.

The current study aimed to describe the environmental presence of pathogenic *Leptospira* spp. in Sydney urban recreational parks in the Australian summer (December 2023–January 2024), test any differences in pathogenic *Leptospira* observations between dog-allowed and dog-prohibited areas, and identify any spatial autocorrelation of the test results. 

## 2. Materials and Methods

### 2.1. Sampling Locations

The study followed a cross-sectional, observational design.

From 13 December 2023 to 17 January 2024, environmental samples were collected from recreational parks managed by local councils in the Inner West and the City of Sydney, Australia [33,34]. The City of Sydney council area is a heavily populated urban area (26.68 km^2^ with 218,096 residents in 2022 [35]), which contains the central business district of Sydney. The Inner West council area is the area immediately west of the City of Sydney, which housed 183,105 persons across 35.22 km^2^ in 2022 [36]. The mean monthly rainfall during the study period was 14 mm, and the mean monthly maximum and minimum temperature were 28.2–28.3 °C and 19.4–20.7 °C, respectively [37].

The list of parks sampled was manually compiled, and only parks with both dog-allowed and dog-prohibited (e.g., playgrounds, food-processing areas, including BBQ grounds) areas were shortlisted. The coordinates (decimal degrees) of the parks were manually extracted from Google Earth (Google, Mountain View, CA, USA) [38]. A shapefile of the study area (council areas of the Inner West and City of Sydney) was accessed from the 2021 Australian Census geopackage (Australian Bureau of Statistics, Canberra, Australia) [39], then processed as a single polygon and visualised using ArcGIS Pro 2.5.0 (ESRI, Redlands, CA, USA) [40]. The polygon was then subdivided into four quadrants of equal area (approximately 15.50 km^2^ per quadrant). Within each spatial quadrant, five parks were tentatively, non-randomly selected from the candidate park list. Substitution of manually selected parks was also conducted upon field observation: only parks where at least one water sample could be collected within either a dog-allowed or dog-prohibited area were included, and parks without open or stagnant water resources were substituted with other parks in the same quadrant. The spatial relationship of parks was also subjectively considered in addition to the inclusion criteria, to ensure the selected parks were relatively homogenously distributed across the study area.

### 2.2. Water and Soil Sampling

For environmental sampling at each park, ideally, one soil sample and one water sample were collected from both dog-allowed and dog-prohibited areas (fenced or open food preparation areas and playgrounds), respectively. If water was not available in both areas, only one water sample was collected. Due to the descriptive nature of the current study, sample collection was conducted without restricting sampling time and under various weather conditions. All environmental samples were stored in sterile specimen containers at ambient temperature (18–30 °C) and transported to the University of Sydney, Camperdown, within four hours post-collection. Prior to DNA extraction and PCR testing, the samples were kept refrigerated at 4 °C.

The targeted water sources in the current study were primarily stagnant water (public dog water bowls, decorative fountains, puddles, water play apparatus, and a pond) to which human and canine visitors have access in the dog-allowed areas, and stagnant water to which human visitors (e.g., children) have access in the dog-prohibited areas. Water samples were also collected from the seashore and human drinking fountains when stagnant water bodies were not observed in the area. From each water body, at least 150 mL of water was collected using sterile pipettes and a glass measuring cup disinfected with 1:100 F10 veterinary disinfectant solution (quaternary ammonium compounds 54.0 g/L) (F10 Products, Loughborough, UK) [41] between sample collections and soaked in the 1:100 F10 solution for at least 20 min on the morning of each collection date. The sea-water samples were collected 10 cm from the seashore of beaches where off-leash dogs or dog footprints were observed on the collection date. The pond water sample was collected 10 cm from the pond edge. All sea and pond water samples were collected from the surface of water bodies (at approximately 5 cm depth).

At least 50 g of soil samples were collected at a depth of approximately 10 cm from open space (e.g., grass lawn, beach) and ground underneath canopy cover or playground mulch. All sampling sites were easily accessible by humans in the dog-prohibited areas, or humans and dogs in the dog-allowed areas. The soil samples were collected using metal trowels disinfected with the 1:100 F10 solution in between sampling sessions and soaked in the same solution for at least 20 min on the morning of each collection date. 

### 2.3. DNA Extraction and Quantitative PCR Testing

DNA extraction and quantitative PCR (qPCR) testing of samples were both performed at the Elizabeth Macarthur Agricultural Institute (EMAI), the state veterinary laboratory for NSW. The Qiagen DNeasy Blood and Tissue Kit [42] was used to extract DNA from water samples as performed by others [43,44], while the Qiagen DNeasy PowerSoil Kit [45] was used to extract DNA from water sediment and soil samples, according to the manufacturer’s instructions (Qiagen Inc., Toronto, ON, Canada). Water samples were centrifuged at 4500× *g* for 10 min, and three samples with a pellet greater than 0.5 cm^3^ underwent the soil extraction procedure using the sediment instead of the water extraction procedure [46]. Of the 75 samples, 37 were spiked with 1 μL of in-house synthetic DNA (10,000 copies) to serve as an internal process control (see below). All DNA extracts were stored at 4 °C prior to qPCR testing, for a maximum of 24 h.

The qPCR assay used is a published protocol that detects an 87-base pair (bp) target within the *rrs* (16S) gene present in pathogenic and intermediate *Leptospira* spp. (subclade P1 and P2) [47,48,49]. TaqMan^TM^ Environmental Master Mix 2.0 (Thermo Fisher Scientific, Waltham, MA, USA), a primer concentration of 10 μM each of forward and reverse primers (F—5′ CCCGCGTCCGATTAG 3′; R—5′ TCCATTGTGGCCGRA/GACAC 3′), a 5 μM probe concentration (5′ CTCACCAAGGCGACGATCGGTAGC 3′), and 2 μL of DNA extract, were included in each reaction. Cycling conditions were a 95 °C denaturation step for 10 min, followed by 45 cycles of 95 °C for 15 s and 60 °C for one minute in a QuantStudio 5 Real-Time PCR machine (Thermo Fisher Scientific).

An in-house internal process control was used to determine if inhibition occurred in any of the qPCR reactions. This required the spiking of samples with a known target DNA sequence during the DNA extraction process. Identical primer concentrations as above and a 10 μM probe concentration were used in a multiplex reaction with the *rrs* target. Inhibition was considered to have occurred in the samples if the internal control had a cycle threshold (Ct) greater than 35. A no-template control (NTC) reaction that contained PCR reagents, but no DNA sample was used to detect the presence of DNA contamination in the reagents.

A sample was considered qPCR positive for *Leptospira* DNA if amplification occurred before 40 cycles. A sample was considered not detected (negative qPCR result) if no amplification occurred before 45 cycles. Samples for which a Ct value between 40 and 45 was recorded were considered inconclusive results. For statistical analyses, inconclusive results were regarded as qPCR negative for *Leptospira* DNA.

A subset of qPCR positive samples was submitted for sequencing (Australian Genome Research Facility, Sydney, Australia) and the Basic Local Alignment Search Tool (BLAST) used in Genbank^®^ to confirm amplicons contained *Leptospira* DNA.

### 2.4. Statistical Tests and Spatial Autocorrelation

As the two extraction methods (water vs. soil/water sediment) resulted in different amounts of DNA extracted, statistical tests were only conducted among the same sample group (i.e., soil vs. soil, water vs. water), using the positive or negative detection of pathogenic *Leptospira* DNA in the sample as a dichotomous variable. Insufficient water sediment samples were tested to perform this comparison with this sample subtype, and therefore all three sediment samples were included in the water sample group. A right-tailed chi-squared test and a one-tailed Fisher’s exact test were performed using Microsoft Excel Version 2403 (Microsoft, Redmond, WA, USA) [50] to detect statistically significant differences in the number of positives between water and soil samples, as well as samples collected from dog-allowed and dog-prohibited areas. 

For preliminary spatial analysis, the continuous results were joined to the collection sites (parks) and mapped using the previously extracted park coordinates in ArcGIS Pro 2.5.0. The Global Moran’s Index was calculated for water and soil samples separately.

## 3. Results

**Figure 1:**
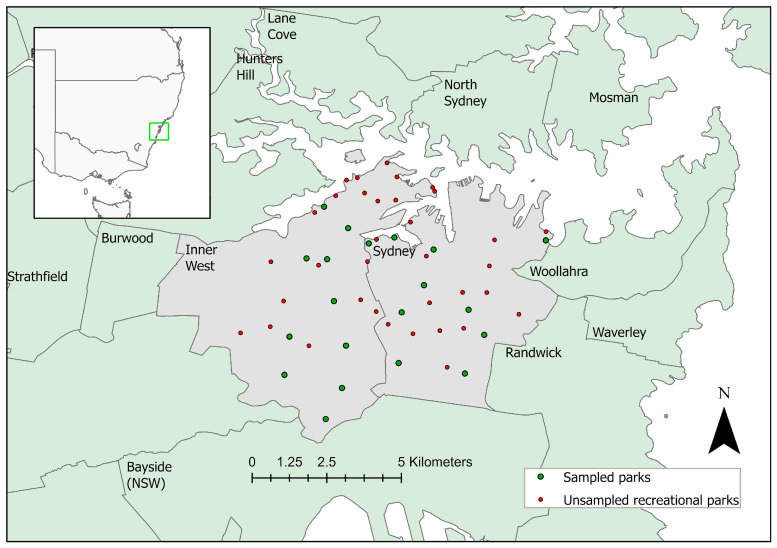
The sampled recreational parks (20 from 55 parks shortlisted) in the council areas of Inner West and City of Sydney (labelled as Sydney in the figure), Australia. Ten parks from each council area were sampled. The green dots indicate sampled parks, and the red dots indicate unsampled parks.

From a shortlist of 55 multipurpose, recreational public parks in the council areas of the Inner West and City of Sydney, Australia, 20 (36%) parks were sampled (Figure 1). Of the 75 environmental samples tested, 35 tested qPCR positive with a Ct ≤ 40, and 22 samples returned inconclusive results (Ct 40–45). The qPCR reaction was negative in 18 samples (no amplification occurred before 45 cycles). Positive qPCR results were observed in either water or soil samples in all 20 parks (Table 1). All qPCR reactions demonstrated an internal process control Ct < 35, indicating there was no PCR inhibition in any of the tested samples.

**Figure 2:**
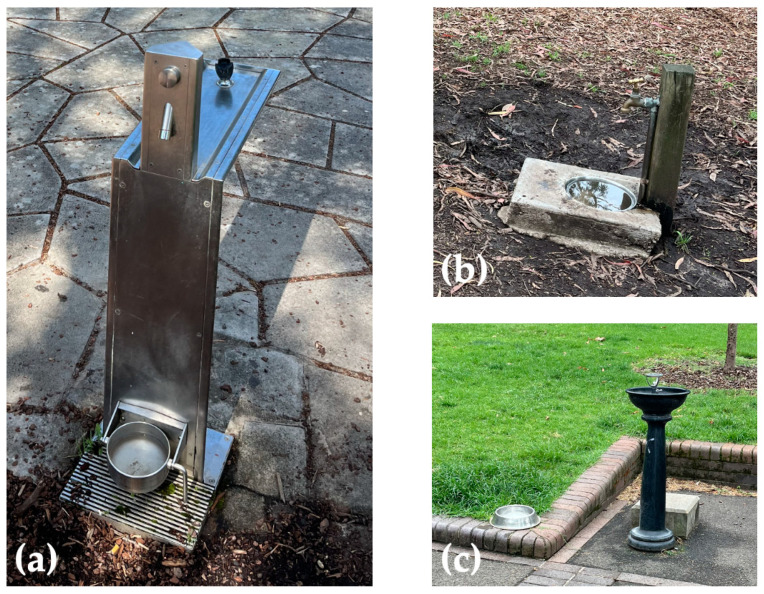
(**a**–**c**). Three types of public dog water bowls were sampled in Sydney urban recreational parks. (**a**) Water bowl as part of the drinking station (*n* = 11); (**b**) water bowl receiving water from a garden tap (*n* = 1); (**c**) water bowl placed and refilled by residents (in this image on the edge of the brick ledge; *n* = 3).

In the 33 water samples, 12 samples (12/33, 36.4%) tested qPCR positive with a Ct range of 28.52–39.10. Thirteen samples were negative, and eight samples returned inconclusive Ct values ranging from 40.19 to 43.64. Among the 12 positive samples, the DNA in 10 samples was extracted using the Qiagen DNeasy Blood and Tissue Kits (Ct 28.52–39.10); 2 samples were prepared from water sediment using the Qiagen DNeasy PowerSoil Kit (Ct 35.20 and 38.51). Table 2 summarises the qPCR results for water samples collected from different locations. Of the 33 water samples tested, 15 were collected from three types of public dog water bowls (Figure 2a–c). None of the samples from the dog water bowls embedded in a drinking station (Figure 2a, *n* = 11) tested qPCR positive, the one water sample from the water bowl illustrated in Figure 2b tested qPCR positive (Ct 38.35), and two of three water samples from dog water bowls that were voluntarily placed by residents (Figure 2c) were qPCR positive (Ct 38.47, 39.10).

In the 42 soil samples, 23 (23/42, 54.8%) tested qPCR positive with a Ct range of 33.78–39.77. Fourteen samples were inconclusive (Ct 40.22–44.02) and five samples yielded no amplification (negative results). It was notable that three soil samples were sand samples, two collected from beaches with off-leash dog areas, and one from a playground (sand pit). One of the beach samples (Ct 37.82) and the sand pit sample (Ct 35.67) tested qPCR positive.

Purified qPCR amplicons from 14 samples yielding the lowest Ct values (Ct < 36) were submitted for sequencing to confirm the presence of Leptospira DNA. Of these 14 samples eight were derived from soil samples and six from water samples. Four soil samples produced results consistent with Leptospira DNA (90.2–100% identity on BLAST alignment). The remaining ten samples did not have discernible sequences, probably due to the low level of target amplicon in the samples. Of the samples that yielded Leptospira sequences, one sample had 100% identity to the species *L. interrogans* and *L. borgpetersonii*, confirming the presence of pathogenic leptospires, two samples yielded sequences with 90.2% and 96.55% identity to *L. wolffii*, which is of intermediate pathogenicity [51,52], and one sample contained sequences with 97.4% identity to *Leptospira* strain WFW58, which was isolated from a waterfall in Thailand and is also considered of intermediate pathogenicity [53]. Sequences were not obtained from any saprophytic leptospires.

Between the two sample groups (water and soil), there was no difference in the overall frequency of positive results (chi-squared test *p* = 0.11 and Fisher’s exact *p* = 0.97). Between the dog-allowed and dog-prohibited areas, chi-squared test (water *p* = 0.2; soil *p* = 0.51) and Fisher’s Exact test (water *p* = 0.95; soil *p* = 0.83) results on the presence of positive qPCR results were both statistically non-significant. For both sample groups, Global Moran’s Index was also non-significant (water Moran’s Index = 0.04, *p* = 0.636; soil Moran’s Index = −0.45, *p* = 0.054).

## 4. Discussion

Despite not being reported in the peer-reviewed literature for over 40 years (1976) in urban Sydney, canine leptospirosis has been considered endemic since December 2017 [32,54]. The current study confirmed the presence of DNA from pathogenic and intermediate pathogenic *Leptospira* spp. in urban Sydney and suggests the ubiquity of pathogenic *Leptospira* in recreational parks across the Inner West and City of Sydney council areas. Our results indicate potential exposure to pathogenic *Leptospira* from drinking water, natural water bodies, and soil in urban recreational areas, although the suspected ubiquity in various environmental media needs to be further confirmed. Thus, while we report the presence of pathogenic *Leptospira* in the parks, the magnitude and virulence of *Leptospira* across the locations require further investigation.

The detection of DNA from pathogenic and intermediate *Leptospira* organisms (P1 and P2) using an *rrs* gene TaqMan assay [47] is consistent with previous environmental surveys where the assay was applied to soil and water samples [48,49]. In Iran [52], Thailand [55], and Peru [5], clinical human leptospirosis and multi-species asymptomatic infections associated with intermediate pathogenic *Leptospira* (e.g., *L. wolffii*) were observed. However, conclusions beyond this observation remain speculative to date, and concerns over the limitations of interpreting the presence of intermediate *Leptospira* in environmental samples have been raised [49]. For this reason, qPCR assays based on the *lipL32* gene of pathogenic *Leptospira* (P1) [56] for environmental leptospiral detection are preferred by some researchers [9,49,57]. Although *rrs* and *lipL32* assays were both originally designed for human clinical samples, there has been no report of false positive detection of saprophytic leptospires using either approach, and the application of both assays in non-cultured environmental samples has been validated [46,49,58]. It is also worth noting that the concentration of *Leptospira* spp. in the samples was relatively low (14/35 positive samples returned Ct < 36). The low leptospiral level likely contributed to the unsuccessful sequencing of ten of the fourteen amplicons, and thus the sequencing outcomes demonstrate the characteristics of environment pathogenic *Leptospira* in Sydney urban parks. We cannot conclude that there was no saprophytic leptospire amplified in the PCR reactions, and the complexity and diversity of bacteria in the environment could have caused an unidentified effect on the results. As amplification inhibition was absent and none of the four sequences acquired from the amplicons were saprophytic *Leptospira*, we are optimistic about the amplification efficiency and sensitivity of the qPCR testing performed. To further confirm the distribution of the two subclades of *Leptospira* and assess the potential spatial heterogeneity, a subsequential, P1-specific assay of the *lipL32* gene should be conducted. Alternatively, a *lipL32* nested-PCR assay to address the low leptospiral concentration [59] or a *rrs* and *lipL32* multiplex assay for higher specificity [60] might be considered.

The four positive sequencing results were sampled from soil, which might suggest that soil is the major environmental reservoir of pathogenic *Leptospira* compared to water in urban Sydney. From water on the metallic surface of a human drinking fountain positioned approximately one metre above the ground, a positive PCR reaction was also detected. This result triggers several questions: is it associated with wildlife contamination, or could it indicate transmission of *Lepstopira* via aerosolized soil in a natural environment? In rats trapped in the City of Sydney council area, 8.1% of the sampled individuals were infected with leptospires and carried the bacteria in their kidneys, which could result in the shedding of the bacteria if individuals are chronically infected [61]. Other common Australian urban wildlife, including flying foxes [62], bandicoots [63], and possums [64], are all potential reservoirs of pathogenic *Leptospira* organisms. It is acknowledged that soil leptospires can inhabit soil for extended periods and contaminate neighbouring water sources [65,66]. Recently, the proliferation of pathogenic *Leptospira* was also observed in waterlogged soil [10]. Considering the unknown interactions between wildlife and environmental reservoirs, together with the long survival of *Leptospira* in soil, understanding the transmission pathways and risk mapping in Sydney can be difficult. It is unrealistic to fully explain the mechanisms underlying the current findings, but investigations into the local wildlife population dynamics should be of high priority to build an overview of the topic.

Regarding the potential role of companion dogs in the transmission of human leptospirosis, we did not find any difference in positive PCR results between dog-allowed and dog-prohibited areas, but we are still far from rejecting the potential canine contribution to the contaminated environment. As legally prescribed by the Companion Animals Act 1998 [67], companion dogs are prohibited within 10 metres of a children’s playground or of a food preparation area. However, the legislation is not properly followed by the public, based on our observations. During sample collection, dogs were observed in a decorative fountain, in BBQ areas, and in an unfenced children’s playground. The urinary shedding of *Leptospira* in Sydney dogs was described in previous clinical canine patients [28], but evidence of asymptomatic shedding in dogs is absent.

Prior to further confirmation, the current results should not be interpreted as indicating the prevalence of pathogenic *Leptospira* in Sydney urban recreational parks. However, the ubiquity of pathogenic *Leptospira* is highly suspected. The high positive rate in urban environments leads to the assumption that human and canine residents are exposed to certain levels of pathogenic *Leptospira* in their daily lives. Chronic, low-dose exposure (e.g., occupational exposure) is often considered a risk factor leading to clinical infection [68,69,70]. Clinical canine and human leptospirosis is not frequently reported in the study areas (eight canine cases reported within or near the Inner West and City of Sydney council areas in 2023). The gap between the suspected ubiquitous exposure and the infection rate further supports the low concentrations of pathogenic *Leptospira* in the environment. Furthermore, one or more of the following factors could be contributing concurrently: (i) low virulence of the *Leptospira* strains in the urban parks; (ii) the unconfirmed spatial or space-time heterogeneity in *Leptospira* distribution [71]; (iii) protective effect provided by the non-mandatory canine vaccination (Protech^®^ C2i containing *L. interrogans* serovar Copenhageni [72]) (a vaccine against *Leptospira* spp. for people is not available in Australia [73]); and (iv) the association between pre-existing conditions or concurrent injuries (e.g., impaired skin barriers [74,75], contacting spiky raspberry plants and soil without skin protection in a recent human leptospirosis outbreak on a farm in rural NSW [76]) and clinical leptospirosis. This study serves as a preliminary attempt to understand and monitor pathogenic *Leptospira* in urban Sydney, and thus it does not reach any level of public health alert. Risks secondary to the environmental *Leptospira* exposure in the study area cannot be evaluated solely from it, but care should be recommended in or near the study area due to the leptospiral presence at the parks, and both medical and veterinary professionals should be informed of the potential risks of contracting leptospirosis from environmental spillover.

As discussed above, the most prominent limitation of this study was the single qPCR assay performed on the samples, which should be strengthened by follow-up testing. Aside from the need for implementing assays for other genes (e.g., *lipL32*), improvement in the DNA extraction methods employed should also be considered. Two commercial DNA extraction kits (DNeasy PowerSoil Kit and DNeasy Tissue Kit) were used in the present study for extraction from soil (and highly sedimented water) and less sedimented water samples, respectively. In contrast to the DNeasy PowerSoil Kit being acknowledged as one of the optimal approaches for DNA extraction from soil samples [46,49,65] and turbid water samples [46], a variety of extraction methods for water samples have been described in the literature. Commercial kits and protocols designed for extracting DNA from blood and tissue (e.g., QIAmp DNA Mini Kit) were used for DNA extraction from environmental surface water samples in Peru [58], and the DNeasy Tissue Kit is a relatively common tool utilised in aquatic biology research [43,44]. In a study in which more turbid, stagnant water samples (e.g., water from animal troughs) were analysed, the QIAmp DNA Stool Mini Kit was used [77]. The DNeasy PowerWater Kit has also been frequently applied to less sedimented water samples (e.g., coastal water, river water, or drinking water) [46,48,78]. Although the DNeasy PowerWater Kit retrieved more DNA from ultrapure water in an experiment in which the performance of several commercial extraction kits on different types of environmental samples was compared, the DNeasy PowerSoil Kit demonstrated excellent efficiency in DNA extraction from sedimented water samples (e.g., pond water, sewage) [46]. The use of the DNeasy Tissue Kit and Dneasy PowerSoil Kit on subtypes of environmental water requires further validation, but trialling the DNeasy PowerSoil Kit on relatively less sedimented water samples (e.g., pond water) is worth considering.

## 5. Conclusions

By analysing 75 environmental samples collected from recreational parks in the council areas of Inner West and City of Sydney, Australia, we conclude that pathogenic *Leptospira* spp. are present and potentially ubiquitous in park environments in urban Sydney. Subsequential PCR assays should be considered to confirm the ubiquity of *Leptospira* in Sydney urban parks. Our initial detection of pathogenic *Leptospira* demonstrates the potential for environmental surveys to be part of longitudinal pathogenic *Leptospira* surveillance strategies and the possible development of a geographically specific risk model.

## Figures and Tables

**Table 1:** The quantitative PCR (qPCR), sequencing and Basic Local Alignment Search Tool (BLAST) results from 75 environmental samples collected from 20 parks in the council areas of Inner West and City of Sydney, Australia (2023–2024 summer). Two parks without adequate water samples available for testing (Park #3 and Park #4) had multiple soil samples collected for testing instead and therefore have more than one result displayed for the same sample type. Samples with a cycle threshold (Ct) value lower than 40 were considered qPCR positive. Samples with Ct values of 40–45 were inconclusive and considered negative for pathogenic *Leptospira* DNA in the statistical analyses. Samples without amplification occurring prior to 45 cycles were considered qPCR negative (i.e., no Ct was recorded). Ct values are recorded in brackets.

No. of Parks (*n* = 20)	Water Results (*n* = 33)		Soil Results (*n* = 42)		Positive Sequencing and BLAST Output (*n* = 4/14)
	Dog-Allowed Area (ct) (Positive *n* = 9/20)	Dog-Prohibited Area (Ct) (Positive *n* = 3/13)	Dog-Allowed Area (Ct) (Positive *n* = 12/20)	Dog-Prohibited Area (Ct) (Positive *n* = 11/22)	
1	**Positive (36.25)**	Negative	Negative	**Positive (39.72)**	
2	**Positive (39.01)**	Inconclusive (43.64)	Negative	Inconclusive (40.58)	
3	Negative	N/A	**Positive (38.81)**	**Positive (37.79)** **Positive (35.77) ^1^**	
4	Negative	N/A	Inconclusive (42.20)	Positive (35.05) ^1,^* Negative	**100% identity to *Leptospira* spp.**
5	**Positive (31.91) ^1^**	N/A	Inconclusive (41.90)	Inconclusive (44.01)	
6	**Positive (38.47)**	**Positive (38.90)**	**Positive (39.51)**	**Positive (36.26)**	
7	Inconclusive (40.56)	Negative	**Positive (35.95) ^1^**	Inconclusive (42.26)	
8	**Positive (32.76) ^1^**	Negative	**Positive (37.82)**	**Positive (37.31)**	
9	**Positive (33.35) ^1^**	N/A	Inconclusive (40.45)	Negative	
10	Inconclusive (43.23)	Inconclusive (43.11)	**Positive (38.49)**	**Positive (38.56)**	
11	Negative	Negative	**Positive (38.12)**	Inconclusive (40.22)	
12	Negative	Inconclusive (40.41)	**Positive (33.95) ^1^**	Inconclusive (42.06)	
13	**Positive (38.51)**	Negative	**Positive (39.77)**	Inconclusive (40.43)	
14	Ngeative	N/A	**Positive (39.52)**	**Positive (37.91)**	
15	Inconclusive (40.41)	Negative	**Positive (36.37)**	Inconclusive (43.44)	
16	Inconclusive (43.59)	N/A	Inconclusive (41.32)	Positive (33.78) ^1,^*	**97.44% identity to *Leptospira* spp.**
17	Negative	**Positive (35.20) ^1^**	Negative	Inconclusive (41.47)	
18	**Positive (39.10)**	Negative	**Positive (34.87) ^1^**	Inconclusive (44.02)	
19	**Positive (38.35)**	**Positive (28.52) ^1^**	Inconclusive (43.02)	Positive (35.67) ^1,^*	**96.55% identity to *Leptospira* spp.**
20	Inconclusive (40.19)	N/A	**Positive (35.14) ^1^**	Positive (35.57) ^1,^*	**90.2% identity to *Leptospira* spp.**

N/A: N/A in Table 1 indicates the unavailability of the specific sample type in the park due to varied park design or climate conditions. Cells in bold represents positive qPCR, sequencing and BLAST results. ^1^ Samples submitted to sequencing. * Sequences returned with positive sequence outcomes.

**Table 2:** The quantitative PCR (qPCR) results from 33 water samples collected from various locations in 20 recreational parks in Sydney (2023–2024 Australian summer). In total, 12/33 (36.4%) water samples tested qPCR positive for pathogenic *Leptospira* DNA. Ct = cycle threshold.

Collection Sites	Number of Positive Samples (%)	Ct Values
Pond	1/1 (100%)	36.25
Seashore	2/2 (100%)	31.91, 32.76
Puddle	3/7 (43.86%)	33.35–38.51
Decorative fountain	1/2 (50%)	39.01
Water play apparatus	1/2 (50%)	28.52
Dog water bowl	3/15 (20%)	38.35–39.10
Human drinking fountain	1/4 (25%)	38.90

## Data Availability

Data are available upon request.
